# Short-Term Impact of Non-Surgical and Surgical Periodontal Therapy on Oral Health-Related Quality of Life in a Greek Population—A Prospective Cohort Study

**DOI:** 10.3390/dj8020054

**Published:** 2020-05-25

**Authors:** Charis Theodoridis, Anastasia Violesti, Maria Nikiforidou, Georgios C. Menexes, Ioannis D. Vouros

**Affiliations:** 1 Department of Preventive Dentistry, Periodontology and Implant Biology, Dental School, Aristotle University of Thessaloniki, 54124 Thessaloniki, Greece; ht_auth.dent@yahoo.com (C.T.); anastasiaviol@gmail.com (A.V.); mnikifor@yahoo.gr (M.N.); 2 Laboratory of Agronomy, School of Agriculture, Aristotle University of Thessaloniki, 54124 Thessaloniki, Greece; gmenexes@agro.auth.gr

**Keywords:** oral health-related quality of life, periodontal disease, non-surgical periodontal therapy, periodontal surgery, OHIP-14, patient-based outcomes

## Abstract

While periodontitis deteriorates patients’ quality of life, non-surgical periodontal treatment seems to offer an improvement. The purpose of the present study was to evaluate the impact of non-surgical and surgical periodontal treatment on the oral health-related quality of life (OHRQoL) utilizing patient-centered assessments and surrogate clinical measurements in Greek adults. Eighty-three individuals with chronic periodontitis were enrolled in the study. Assessment of OHRQoL with the use of the Oral Health Impact Profile (OHIP-14) questionnaire in conjunction with clinical measurements of pocket probing depth (PPD), plaque index (PI) and bleeding on probing (BOP) were performed at baseline (t0), after non-surgical therapy (t1) and after periodontal surgery (t2). A statistically significant reduction of OHIP-14 score was recorded at t1 and t2 examination compared to baseline (*p* < 0.001) and a statistically significant improvement in all clinical parameter at all time points was recorded (*p* < 0.05). No correlation between the clinical parameters and the total score of OHIP-14 was recorded at any time point. Non-surgical periodontal treatment seemed to improve OHRQoL in terms of OHIP-14 scores, whilst supplementary surgical periodontal therapy did not offer any additional benefit. No correlation was found between patients’ perception of quality of life expressed by OHIP-14 score and the surrogate clinical parameters.

## 1. Introduction

During the last decades, research on periodontal health and pathology has been based on a biomedical approach to disease. The clinical signs of chronic periodontitis, such as clinical attachment loss, probing pocket depth and bleeding on probing have been overemphasized by the clinicians, as they provide evidence on both disease severity and effectiveness of periodontal therapy but offer little insight into more subjective patient-based outcomes, such as quality of life.

According to the World Health Organization (WHO) [1], quality of life (QoL) is defined as individuals’ perception of position in life in the context of the culture and value system in which they live and in relationship to their goals, expectations, standards and concerns. The term ‘oral health-related quality of life’ (OHRQoL) characterizes an individuals’ perception of how oral health conditions impact on the overall QoL [2,3,4].

From a biopsychosocial perspective, disease management has to consider social, psychological, and behavioral dimensions [5] and hence, periodontal disease cannot only affect the patient’s speech vocalization and eating capacity, but also daily activities, interpersonal relationships and, generally, their QoL. In line with that perspective, there is a trend lately, when designing a study, to consider the patients’ needs and perception of therapy. This methodological approach characterized as a patient-centered approach, has been utilized by a number of studies focusing on the patients’ expectations of therapy. Patient-centered approaches to periodontal disease can lead to a broader understanding of the disease’s effects and in this context, they are obviously associated with Oral health conditions, which in turn have an impact on quality of life. Furthermore, the improvement of patient-centered outcome may be more rewarding for the clinician [6].

Patient-centered outcomes have been recently addressed in the literature and a number of studies have investigated the impact of periodontal disease and different therapeutic modalities on the well-being of patients [7,8,9,10,11,12]. With reference to them, while periodontal disease was associated with a negative impact on OHRQoL [13,14,15,16,17,18], initial periodontal therapy was associated with an improvement [19,20,21,22,23,24,25,26]. In terms of surgical periodontal treatment, there is a limited number of studies evaluating the influence of periodontal surgery indicating that it has a lesser effect on QoL [12,27,28,29,30]. Additionally, taking into account that different populations may be characterized by different cultural and behavioral aspects, QoL alterations after periodontal therapy may vary among different nations or geographic ranges. Therefore, more studies are needed to assess the effect of surgical periodontal therapy for different surgical therapeutic modalities and the effect of initial periodontal therapy on the QoL of different populations, which have yet to be studied.

Based on the insights above, the aims of the present study were to evaluate the early post-treatment impact of non-surgical and surgical periodontal treatment on the OHRQoL in periodontal patients by using the Oral Health Impact Profile (OHIP-14) [31] and to investigate any associations between the QoL and clinical parameters, in a Greek population. A null hypothesis (H0) that there is no significant difference between pre- and post-treatment OHIP-14 scores, regarding both non-surgical and surgical periodontal therapy, was considered.

## 2. Materials and Methods

### 2.1. Study Population

The present cohort comprised 83 consecutive chronic periodontitis patients who were referred for treatment to the Department of Preventive Dentistry, Periodontology and Implant Biology, Dental School of Aristotle University of Thessaloniki. The patients were recruited from November 2016 to March 2018.

The following inclusion criteria were adopted: (1) diagnosis of moderate to severe chronic periodontitis, (2) smokers < 10 cigarettes daily, or non-smokers, (3) fully dentate or partially edentulous patients with at least 18 natural teeth.

Exclusion criteria were: (1) age younger than 18 years, (2) presence of uncontrolled systemic disease or systemic diseases with symptoms and pathological lesions in oral cavity, (3) history of neoplasm and radiotherapy in the maxillofacial area in the previous five years, (4) pregnant or lactating women, (5) obese patients with BMI more than 30, (6) history of non-surgical or surgical periodontal treatment in the previous 12 months.

The patients enrolled were presented with a moderate to severe chronic periodontitis according to the AAP Classification in regards to severity. Their periodontal condition was categorized on the basis of clinical attachment loss (CAL) as mild periodontitis, CAL 1–2 mm, moderate periodontitis, CAL 3–4 mm and severe periodontitis, CAL ≥ 5 mm. [32,33].

### 2.2. Ethical Considerations

An approval from an ethics committee has been obtained prior to research start. More specifically, all subjects gave their informed consent for inclusion before they participated in the study. The study was conducted in accordance with the Declaration of Helsinki, and the protocol was approved by the Ethics Committee of Faculty of Dentistry, School of Health Science, Aristotle University Thessaloniki (Project identification code: 3/10-10-2013). 

### 2.3. Study Design

**Figure 1:**
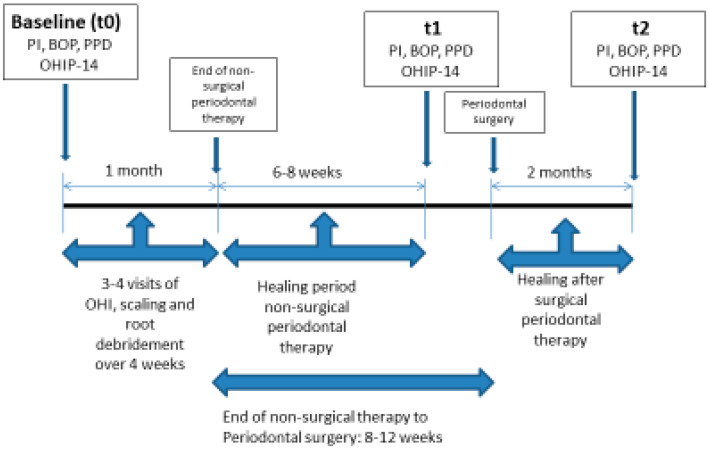
Study flow chart

Figure 1 summarizes the flow of the current study.

- The medical and dental histories of the participants were obtained in order to meet the inclusion criteria. Socio-demographic data were also collected (age, gender, marital status, place of residence, years of education, family income per month, occupation, smoking habits, frequency of brushing, use of dental floss and frequency of previous dental visits).
- Assessment of oral health-related quality of life with the use of OHIP-14 questionnaire was performed:
  1. At baseline (t0), before initial periodontal treatment
  2. At phase I (t1), 6–8 weeks after non-surgical periodontal treatment
  3. At phase II (t2), 8 weeks after surgical periodontal treatment
- Measurement of clinical parameters: The following periodontal clinical parameters were recorded at six sites per tooth, mesio-buccal, buccal, disto-buccal and mesio-lingual, lingual, disto-lingual; probing pocket depth (PD), Plaque Index (PI) and Bleeding On Probing (BOP). Clinical examination was performed using a periodontal probe (Hu-Friedy XP-23/QW, Hu-Friedy, Chicago, IL, USA), that was placed parallel to the long axis of each tooth; recordings were assessed to the nearest millimeter. The presence or absence of bleeding on probing was calculated as Full Mouth Bleeding scores (FMBS) [34] and the presence or absence of supragingival dental plaque was recorded using the O’Leary Plaque Control Record [35] expressed as percentages. Both the above indices were applied to evaluate patient compliance as well, with desired compliance being <20%. Probing pocket depth was measured and then expressed in the study as the proportion of the sites showing probing pocket depth ≥ 5 mm. The clinical examinations at each time point were performed by one trained examiner (A.V.). The assessment of intra-examiner reproducibility for the PPD, PI and BOP examinations was performed by double recordings in 20 participants. The intraclass correlation coefficients for intra-examiner reproducibility was 0.83 for PPD, 0.89 for PI and 0.91 for BOP.

### 2.4. Data Collection

The Greek short-form version of the Oral Health Impact Profile (OHIP-14) was used to evaluate the impact of periodontal disease on OHRQoL [2,31,36]. It constitutes one of the most comprehensive instruments available in the literature for the purpose of detecting dysfunction, discomfort and disability attributed to oral conditions. Its psychometric properties, validity and reliability have been widely assessed with satisfactory results [31,35]. Furthermore, the Greek version has been applied in a study evaluating the impact of Oral Health on the Quality of life of complete denture wearers in a Greek population. The authors of the study utilized the OHIP-14 questionnaire, which was adapted into Greek using the method of back translation and was found to be reliable and valid [37]. Another more recent trial confirmed the above findings in an adult Greek population [38].

The OHIP-14 is a patient-centered questionnaire that measures OHRQoL using 14 items to capture measures of seven dimensions: functional limitation (F1), physical pain (F2), psychological discomfort (F3), physical disability (F4), psychological disability (F5), social disability (F6) and handicap (F7). These seven domains were derived from the oral health model described by Locker et al. [39]. Each dimension was measured by two questions. Patients were asked how often they had experienced negative impacts in these dimensions during the last year. Responses to the items were recorded on a five-point Likert scale: 0, never; 1, hardly ever; 2, occasionally; 3, fairly often; 4, very often. The overall score for OHIP-14 was obtained by summing all responses and thus ranged from 0 (no problems at all) to 56 (all issues experienced very often).

### 2.5. Procedures

Before the initial periodontal therapy, patients were asked to respond to the first OHIP-14 questionnaire (t0). Non-surgical periodontal therapy included oral hygiene instructions, supra-gingival scaling and subgingival scaling and root planing performed with the use of 3/4, 11/12 and 13/14 Gracey curettes (Hu-Friedy, Chicago, IL, USA) in conjunction with ultrasonic devices (EMS, Nyon, Switzerland on a quadrant-base under local anesthesia. As a result, non-surgical periodontal therapy was completed in four sessions.

After 6–8 weeks of tissue healing, clinical re-evaluation was performed and patients were asked to respond for a second time to the OHIP-14 questionnaire (t1). According to the clinical findings of the re-evaluation, in cases presented with residual probing pocket depth ≥ 6 mm proceeding to surgical access therapy was considered. Periodontal surgery was conducted 8–12 weeks after completion of phase I periodontal therapy, in participants, who presented with at least one site with a residual probing pocket depth ≥ 6 mm and simultaneous bleeding on probing with adequate oral hygiene levels (full-mouth bleeding and plaque scores ≤ 20%). Depending on the case requirements as well as the anatomy of periodontal tissues (periodontal biotype, biological width) a flap modality was chosen, the modified Widman flap was performed in order to gain access for further removal of the subgingival deposits [40] whereas a very conservative version of apically positioned flap in conjunction with osteoplasty only, was selected for pocket depth reduction/elimination.

Firstly, for both the above mentioned periodontal surgeries, an inter-radicular incision parallel to the long axis of the operated teeth at buccal and lingual sites was performed, including the interproximal surfaces and extending to the alveolar bone using the Bard Parker knife with blade No 15c. Vertical releasing incisions were avoided. A full thickness mucoperiosteal flap was then reflected using periosteal elevators, which extended beyond the mucogingival junction only during the apically repositioning flap surgery. A thorough debridement and removal of granulation tissue and hard deposits was implemented, while bone architecture was contoured only if necessary. The modified Widman flap was repositioned to its natural position and secured in place using interrupted simple sutures in order to obtain primary closure of the interdental space. The apically positioned flap was secured in the new apical position by continuous sling sutures.

Non-steroidal anti-inflammatory medication was administered twice/daily to the patients for the first five days after the surgery and chlorhexidine digluconate 0.2% for a week, until suture removal. No systemic antibiotics were administered. Periodontal treatment was performed by four postgraduate students of the Department of Periodontology, who were blinded to the present study purpose, inclusion criteria and design.

After a period of two months of healing, the patients were re-evaluated and completed for the third time the OHIP-14 questionnaire (t2).

### 2.6. Statistical Analysis

Sample size estimation was carried out based on our pilot study and on previous large epidemiological studies. More specifically, mean OHIP-14 score (24.19) and SD (7.04) from patients suffering from advanced periodontitis [8], as well as mean OHIP-14 score (14.7) and SD (11) from a Greek community metropolitan adult population [41], were taken into account. For α = 0.05 and power = 80%, a total sample of a minimum of 11 subjects is needed, in order for a two-tailed test to be conducted, while for power = 95%, a minimum of 17 subjects are required. The sample size was calculated with G*Power v.3.1.9.2 (Frantz Faul, Universität Kiel, Germany).

Data were summarized by computing absolute (counts) and relative frequencies (percentages %), measures of central tendency (mean and median values), measures of variability (minimum and maximum values, standard deviations (SD)) and correlation indices (Spearman’s rho). Comparisons of the distributions, mainly relative to their central tendency, of patients’ clinical parameters and their total OHIP-14 score among the three time points (t0, t1 and t2) were accomplished with the Wilcoxon’s signed-rank test. In all hypothesis testing procedures (Wilcoxon test and significance test of Spearman’s rho rank correlation coefficient), the observed significance level (*p*-value) was computed by means of Monte-Carlo simulation method [42] based on 10,000 random samples. This method leads to safe inferences even in cases where the methodological presuppositions of the non-parametric tests are not fulfilled (random samples, independent measurements, symmetrical distributions and absence of heavy outliers). Non-parametric statistical tests were considered more appropriate for analyzing the data of this study, since the normality and other methodological assumptions were not satisfied. In all hypothesis testing procedures the significance level was predetermined at a = 0.05 (or *p* ≤ 0.05). Statistical analyses were done with the SPSS v.15.0 software (SPSS Inc., Ill, Chicago, IL, USA) enhanced with the module Exact Tests (for the implementation of Monte-Carlo simulation method).

## 3. Results

A total of 83 Caucasian individuals were initially recruited, eight of whom dropped out while 75 completed the study. Inability to attend the recall appointments and health issues were the reasons for dropouts. Thirty-one of the 75 participants underwent surgical periodontal surgery as well. The demographic characteristics of participants are presented on Table 1

The mean total score of the OHIP-14 questionnaire at baseline (t0) was 16.33. After initial periodontal treatment (t1) it was reduced to 11.96, while the respective value following surgical periodontal treatment was found to be 11.06 (t2) (Table 2). A statistically significant reduction of OHIP score was recorded at the t1 examination compared to the t0 examination (*p* < 0.001), as well as between t0 and t2 (*p* < 0.001). However, the difference in total score between time points t1 and t2 was not found to be statistically significant (*p* = 0.441).

The seven subdomains of the questionnaire correspondent to functional limitation, physical pain, psychological discomfort, physical disability, psychological disability, social disability and handicap taken at baseline (t0) (Total score t0), at phase I (Total score t1) and at phase II (Total score t2) are presented in Table 3. A statistically significant difference in the scores was recorded between t0 and t1 as well as t0 and t2 for all subdimensions except for psychological disability and social disability.

The changes in the clinical assessment of probing pocket depth, expressed as the proportion of sites showing probing pocket depth ≥ 5 mm (PD), plaque index (PI) and bleeding on probing (BOP) at the three time points, t0, t1 and t2, are presented on Table 4.

Differences in all clinical parameters were found to be statistically significant between all time points (in all statistical comparisons *p* < 0.05) but were not statistically significant correlated with the total score of the OHIP-14 questionnaire at all three time points (Table 5). Examining the data presented in Table 5, none of the Spearman’s rank correlation coefficients were statistically and clinically significant (all *p*-values were greater than 0.15, in addition, all correlation coefficients were less than 0.21, a value that corresponds in general to weak association).

## 4. Discussion

Periodontal disease is a condition that undoubtfully affects patients’ well-being and QoL but only a few studies have reported on the effects of mechanical non-surgical periodontal treatment in conjunction with periodontal surgery on the OHRQoL of periodontally compromised patients by using Oral Health Impact Profile (OHIP-14). In the present study, a statistically significant reduction in the total score of the OHIP-14 after initial cause related therapy was observed, which indicates that initial mechanical treatment produced a positive impact on the OHRQoL and thus H0 may be rejected. This positive impact was not further improved by additional periodontal surgical treatment. It seems that patients perceive an improvement of their OHRQoL after initial therapy, whereas surgical treatment does not seem to significantly favor this perception. This enhances the importance of initial non-surgical approach as the cornerstone of therapy as it not only improves clinical parameters but has also a positive impact on a patient’s perception of treatment outcomes. A non-surgical treatment, in terms of surrogate periodontal parameters, was found to achieve comparable results to surgical approaches in the long term [43,44,45,46]; clinicians should consider that application of surgical treatment could be limited solely to specific and selected cases of periodontal lesions.

As indicated above, information concerning the effect of surgical periodontal therapy on quality of life is limited. Oczelik et al. [27] compared three therapeutic modalities, non-surgical periodontal therapy, surgical periodontal therapy, and surgical regenerative treatment with the use of enamel matrix derivative (EMD) application. Non-surgical therapy and regenerative surgical therapy utilizing EMD showed better results regarding the OHRQoL than surgical therapy alone, although this improvement and in general the findings of this study were limited only to the first week postoperatively. The effect of periodontal surgery on quality of life was studied by Chou et al. [30], who reported a significant improvement after a surgical approach to periodontal defects. A more favorable result was reported for regenerative surgery in comparison to respective surgery, although this study did not evaluate the effect of initial periodontal therapy on the quality of life.

Saito et al. [28] followed up 21 patients that received initial periodontal therapy in conjunction with periodontal surgery. Non-surgical periodontal therapy was found to improve OHRQoL (phase I) but no statistically significant further improvement was observed after surgical treatment (phase II). This is in accordance with the findings of the present study that question the efficacy of periodontal surgery when patients’ perceptions of treatment outcome are taken into consideration as the endpoint of therapy. Although, these results come from a pilot study with a small sample size and should be interpreted with caution, especially when relatively small mean differences are noted, the same research group, confirmed the absence of a statistically significant improvement of QoL from phase I to phase II in a larger and more recent study [29].

Another noteworthy finding of our study is that no correlation was recorded between the OHIP-14 scores and the clinical periodontal variables at all three time points. This finding is consistent with the findings of other relevant studies [20,29], though one study noted an association between low OHRQoL scores and reductions of deep periodontal pockets (PD > 4 mm) after periodontal surgery [26]. This means that the perception of patients regarding their wellbeing is not related to the clinicians’ objective assessment expressed as surrogate clinical parameters.

Taking into account that there is a heterogeneity in both the instruments used and the inclusion criteria regarding periodontitis cases among studies investigating patients’ perceptions after periodontal therapy, researchers should be skeptical when discussing the potential impact of various periodontal approaches to OHRQoL and when interpreting patient-centered outcomes.

Given the significance of patients’ perceptions of periodontal disease as well as periodontal care on quality of life, there is a need for the addition of patient-centered outcomes further to the use of clinical surrogate indexes when designing clinical studies. Patient-centered outcomes are significant when evaluating a clinical condition and should be included in clinical research. Not only reporting of these outcomes accords with evidenced-based approach [47] but also, their improvement can motivate patients towards a more beneficial oral health behavior, which is crucial for the successful management of periodontal disease in the long term [12,27]. In this way, clinicians can achieve a better understanding of the problems that patients affected by periodontal diseases face, and can focus accordingly on more appropriate treatment planning, embracing a holistic perception of the underlying ailment.

To the best of our knowledge, this study is the only one that enrolls population from Southern Europe and investigates OHIP-14 scores before and after periodontal therapy. This fact may be substantial if WHO definition of QoL is considered, which incorporates culture and patients’ environment factors, [1] while indicating that the subjective perception of QoL may be influenced differently among different geographical and cultural aspects. This probable variation of OHRQoL among different countries and regions, has been pointed out in previous studies as well [41,48] and, consequently, researchers recognized the importance of OHRQoL comparisons between different countries [48]

Our cohort ended up with 75 patients, out of 83, that completed phase I, 31 of whom were assessed after surgical treatment as well (phase II). This sample size leads to a satisfactory power which may be considered sufficient for assessing the aforementioned mean differences (t0–t1 and t0–t2). Nevertheless, we could state that the heterogeneity of different surgical approaches, primarily due to individualized treatment planning, when periodontal surgery was applied, might be considered a study limitation. This limitation, which has been noted in previous studies in the literature as well, combined with the fact that patients who undergo periodontal surgery often present more advanced periodontal lesions compared to participants treated with NST only, may influence the outcome comparability between phase I and II. Therefore, even though there has not been a detection of any significant alteration towards further improvement in OHRQoL after periodontal surgery, a different study design, which incorporates a larger surgery cohort, may be more suitable to assess relatively small differences in OHRQoL, like the ones that are being observed between phase I and II. Another future research direction could be the evaluation of any probable impact of periodontal therapy on more general aspects of QoL, expanding the hypothesis beyond oral health. Considerable evidence regarding periodontal treatment relationship with short-term systemic inflammation and endothelial dysfunction [49], as well as more recent findings, which connect periodontal inflammation with specific biomarkers, such as Endothelin-1, Asymmetric Dimethylarginine (ADMA) and vitamin D [50,51,52], may justify such a perspective.

## 5. Conclusions

- Periodontal disease negatively affects the oral health-related quality of life and periodontal therapy has been beneficial from a patient-centered point of view in Greek adults.
- There is no correlation between the patients’ perception of quality of life expressed by OHIP-14 score and the surrogate clinical parameters assessed by the clinicians.
- The key message, according to the findings of the study, is that non-surgical periodontal treatment significantly improves function, aesthetics and psychological aspects of periodontal patients, while surgical therapy does not seem to offer any additional benefit in all the above aspects.

## Figures and Tables

**Table 1:** Distribution of demographic characteristics of participants (N = 75).

Demographic	Count (%)
*Gender*	
Male	27 (36%)
Female	48 (64%)
*Marital Status*	
Never married	13 (17.3%)
Married	50 (66.7%)
Separated/divorced	7 (9.3%)
Widowed	5 (6.7%)
*Education status*	
Primary	7 (9.3%)
Secondary	16 (21.3%)
Tertiary (non-degree)	28 (37.3%)
University degree	23 (30.7%)
Student	1 (1.3%)
*Family income (Euro/year)*	
<6.000 EUR	13 (17.3%)
6.000–11.999 EUR	23 (30.3%)
12.000–17.999 EUR	19 (25.3%)
18.000–23.999 EUR	1 (1.3%)
>24.000 EUR	1 (1.3%)
*Smoking (<10 cigarettes)*	
Yes	35 (46.7%)
No	40 (53.3%)
*Systemic disease*	
Yes	34 (45.3%)
No	41 (54.7%)
*Daily use of toothbrush*	
Yes	60 (80%)
No	15 (20%)
*Daily use of interdental cleaning methods*	
Yes	32 (42.7%)
No	43 (57.3%)
*Frequency of prophylaxis visits yearly*	
None	28 (37.3%)
Once	30 (40%)
Twice	12 (16%)
More	5 (6.7%)
Total	75 (100%)

**Table 2:** Descriptive statistical indices and comparisons of the total scores of the OHIP-14 questionnaire at baseline (Total score t0), at phase I (Total score t1) and at phase II (Total score t2) of the study.

	Min	Median	Max	Mean *	SD **	N
Total score t0	0	16	40	16.33 a	7.91	75
Total score t1	3	11	35	11.96 b	5.89	75
Total score t2	1	11	27	11.06 b	5.48	31

* Mean values followed by different boldface letter are statistically significant different (*p* ≤ 0.05) according to a series of Wilcoxon tests. ** SD: Standard Deviation.

**Table 3:** Descriptive statistical indices and comparisons of the total scores of the seven dimensions of the OHIP-14 questionnaire at baseline (t0), at phase I (t1) and at phase II (t2) of the study.

OHIP-14		Min	Median	Max	Mean *	SD **	N
Functional limitation	F1 (t0)	0	2	8	2.7 **a**	2.2	75
	F1 (t1)	0	2	7	1.6 **b**	1.4	75
	F1 (t2)	0	1	7	1.5 **b**	1.7	31
Physical pain	F2 (t0)	0	3	8	3.6 **a**	2.0	75
	F2 (t1)	0	2	7	2.5 **b**	1.5	75
	F2 (t2)	0	2	5	2.2 **b**	1.2	31
psychological discomfort	F3 (t0)	0	1	7	1.7 **a**	1.9	75
	F3 (t1)	0	1	4	1.0 **b**	1.1	75
	F3 (t2)	0	1	4	1.0 **b**	1.1	31
Physical disability	F4 (t0)	0	3	7	2.7 **a**	1.8	75
	F4 (t1)	0	2	5	2.1 **b**	1.5	75
	F4 (t2)	0	1	7	1.8 **b**	1.8	31
Psychological disability	F5 (t0)	0	2	8	2.2 **a**	1.9	75
	F5 (t1)	0	1	7	1.3 **b**	1.4	75
	F5 (t2)	0	1	4	1.3 **b**	1.0	31
Social disability	F6 (t0)	0	0	6	0.9 **a**	1.9	75
	F6 (t1)	0	0	4	0.7 **a**	1.4	75
	F6 (t2)	0	0	2	0.5 **a**	1.0	31
Handicap	F7 (t0)	0	2	7	2.3 **a**	1.7	75
	F7 (t1)	0	1	7	1.6 **b**	1.4	75
	F7 (t2)	0	1	6	1.3 **b**	1.4	31

* For each dimension of the OHIP-14 questionnaire (F1 to F7), mean values followed by different boldface letter are statistically significant different (*p* ≤ 0.05) according to a series of Wilcoxon tests. ** SD: Standard Deviation.

**Table 4:** Probing pocket depth (proportion of sites with PD ≥ 5 mm) (PD), plaque index (PI) and bleeding on probing (BOP) at baseline (t0), at phase I (t1) and at phase II (t2) of the study.

	Min	Median	Max	Mean*	SD **	N
PD (t0)	0.044	0.317	0.887	0.364 **a**	0.218	75
PD (t1)	0.007	0.130	0.607	0.184 **b**	0.142	75
PD (t2)	0.000	0.097	0.413	0.121 **c**	0.100	31
PI (t0)	0.154	0.500	1.000	0.560 **a**	0.243	75
PI (t1)	0.047	0.219	0.535	0.229 **b**	0.108	75
PI (t2)	0.060	0.121	0.296	0.135 **c**	0.057	31
BOP (t0)	0.106	0.593	1.000	0.606 **a**	0.230	75
BOP (t1)	0.061	0.237	0.675	0.241 **b**	0.122	75
BOP (t2)	0.040	0.115	0.333	0.136 **c**	0.075	31

* For each clinical parameter (PD, PI, and BOP), mean values followed by different boldface letter are statistically significant different (*p* ≤ 0.05) according to a series of Wilcoxon tests. ** SD: Standard Deviation.

**Table 5:** Correlation (Spearman’s rho) between probing pocket depth (PD), plaque index (PI) and bleeding on probing (BOP) and the corresponding total Scores of the OHIP-14 questionnaire at baseline (t0), at phase I (t1) and at phase II (t2) of the study.

	Base Line (t0)	Phase I (t1)	Phase II (t2)
	PD (t0)	PD (t1)	PD (t2)
Total OHIP-14 score at t0	rho = 0.167, *p* = 0.153	rho = 0.048, *p* = 0.685	rho = 0.210, *p* = 0.256
	PI (t0)	PI (t1)	PI (t0)
Total OHIP-14 score at t1	rho = 0.028, *p* = 0.808	rho = 0.091, *p* = 0.437	rho = 0.095, *p* = 0.611
	BOP (t0)	BOP (t1)	BOP (t2)
Total OHIP-14 score at t2	rho = 0.145, *p* = 0.216	rho = 0.141, *p* = 0.227	rho = 0.146, *p* = 0.434
